# Faricimab for Diabetic Macular Edema in Eyes Vitrectomized for Proliferative Diabetic Retinopathy: A 12-Month Retrospective Study

**DOI:** 10.3390/jcm15145370

**Published:** 2026-07-09

**Authors:** Kyunga Yoon, Ayumi Usui-Ouchi, Yoshihito Sakanishi, Nobuyuki Ebihara, Shintaro Nakao

**Affiliations:** 1 Department of Ophthalmology, Juntendo University Urayasu Hospital, Urayasu 279-0021, Chiba, Japan; 2 Department of Ophthalmology, Juntendo University Hospital, Bunkyo, Tokyo 113-8421, Japan

**Keywords:** faricimab, diabetic macular edema, vitrectomized eyes, proliferative diabetic retinopathy, angiopoietin-2, VEGF, bispecific antibody, intravitreal injection, real-world evidence

## Abstract

**Background/Objectives:** Faricimab is a novel bispecific antibody simultaneously inhibiting vascular endothelial growth factor-A (VEGF-A) and angiopoietin-2 (Ang-2). Its efficacy in vitrectomized eyes with diabetic macular edema (DME), a setting with altered intravitreal pharmacokinetics, remains poorly characterized. We evaluated the 12-month outcomes of intravitreal faricimab (IVF) for DME in eyes vitrectomized for proliferative diabetic retinopathy (PDR). **Methods:** We retrospectively reviewed 12 consecutive eyes of 11 patients (mean age 59.4 ± 10.5 years) with DME after pars plana vitrectomy (PPV) for PDR who received IVF (6 mg/0.05 mL) between September 2022 and August 2024 with at least 12 months of follow-up. Best-corrected visual acuity (BCVA, logMAR) and central retinal thickness (CRT) were assessed at baseline (BL), 6 months (M6), and 12 months (M12). **Results:** Eight eyes were treatment-naïve and 4 had been switched from prior anti-VEGF therapy. Two eyes (16.7%) required a change in therapy: one for intraocular inflammation and one for inadequate anatomical response. In the 10 eyes that continued IVF, the mean number of injections was 4.1 ± 1.9. CRT decreased significantly from 489.5 ± 95.9 µm at BL to 328.5 ± 74.7 µm at M6 (*p* = 0.0065) and 307.0 ± 64.3 µm at M12 (*p* = 0.0023; repeated-measures ANOVA with Dunnett’s post hoc test). Mean BCVA improved from 0.33 ± 0.26 to 0.23 ± 0.21 logMAR at M12 (*p* = 0.0894). **Conclusions:** In this small retrospective study of vitrectomized eyes with DME after PPV for PDR, IVF was associated with significant anatomical improvement, while visual acuity remained stable over 12 months, with a relatively low injection burden. Faricimab may be a useful therapeutic option in this challenging population, although larger prospective studies are warranted to confirm these findings.

## 1. Introduction

Diabetic macular edema (DME) is a leading cause of vision impairment in patients with diabetic retinopathy, and intravitreal anti-vascular endothelial growth factor (anti-VEGF) therapy has become the standard of care [1]. Faricimab is a novel bispecific antibody that simultaneously neutralizes VEGF-A and angiopoietin-2 (Ang-2), two key drivers of vascular instability and inflammation in DME. In the pivotal phase III YOSEMITE and RHINE trials, faricimab achieved non-inferior visual acuity gains compared with aflibercept, with a higher proportion of patients eligible for extended dosing intervals and superior anatomical drying [2], and consistent efficacy, durability, and safety have been confirmed in the YOSEMITE Japan subgroup over 2 years [3]. Long-term efficacy, durability, and safety of faricimab have been further confirmed in the RHONE-X 4-year extension study [4].

Eyes that have previously undergone pars plana vitrectomy (PPV) for proliferative diabetic retinopathy (PDR) represent a clinically distinct and challenging population. Persistent or recurrent DME is a recognized long-term complication after PPV for PDR, and represents a particularly difficult clinical scenario, as the underlying retinal disease is typically advanced at the time of surgery. With the advent of small-gauge minimally invasive vitrectomy and improved postoperative visual outcomes, persistent postoperative DME has become an important determinant of long-term visual prognosis in this population. In a Japanese cohort of 124 PDR eyes treated with PPV at Yamagata University Hospital, postoperative DME has been reported in approximately 8% of eyes [5,6]. Such eyes typically present with advanced diabetic retinopathy, often long-standing diabetes, and a spectrum of systemic comorbidities including renal impairment, all of which can complicate management. Furthermore, removal of the vitreous gel accelerates intravitreal drug clearance and shortens drug half-life, which has been associated with reduced anti-VEGF durability and an increased treatment burden in real-world practice [7,8].

Despite the clinical importance of this scenario, real-world data on faricimab in vitrectomized eyes with DME remain remarkably scarce. Most large-cohort and long-term real-world studies of faricimab for DME have either excluded eyes with prior vitrectomy or have not separately analyzed this subgroup [9,10,11,12], and the only existing data in vitrectomized eyes consist of a small subgroup analysis within a mixed cohort [8] and a single case report in neovascular age-related macular degeneration [13]. Long-term dedicated outcome data in this population are therefore lacking. Faricimab has a higher molecular weight than aflibercept (~150 kDa vs. 115 kDa) and a longer simulated intravitreal half-life, theoretical properties that could be advantageous in vitrectomized eyes [14]. Here, we report the 12-month outcomes of intravitreal faricimab (IVF) in a consecutive series of vitrectomized eyes with DME after PPV for PDR.

## 2. Materials and Methods

### 2.1. Study Design and Ethics

This single-center retrospective study was conducted at the Department of Ophthalmology, Juntendo University Urayasu Hospital, Urayasu, Japan. The study adhered to the tenets of the Declaration of Helsinki and was approved by the Institutional Review Board of Juntendo University Urayasu Hospital. Because of the retrospective design, the requirement for written informed consent was waived; an opt-out option was provided through the institutional website.

### 2.2. Patients

We reviewed consecutive patients who initiated IVF for DME in vitrectomized eyes from 16 September 2022 (the date of IRB approval) through August 2024 and who completed at least 12 months of follow-up. Eligibility required: (i) prior PPV for PDR; (ii) center-involving DME with central retinal thickness (CRT) ≥ 300 µm on spectral-domain optical coherence tomography (SD-OCT); (iii) at least one IVF injection; and (iv) availability of clinical and imaging data at baseline (BL), 6 months (M6), and 12 months (M12). Exclusion criteria were concomitant retinal pathology unrelated to diabetic retinopathy, intraocular surgery within 3 months prior to IVF (other than the index PPV), and incomplete follow-up. The index PPV procedures had been performed using 25- or 27-gauge transconjunctival sutureless vitrectomy with core vitrectomy, posterior hyaloid removal, segmentation and delamination of fibrovascular proliferative tissue, and supplemental panretinal photocoagulation.

### 2.3. Treatment Protocol

All eyes received intravitreal faricimab (Vabysmo^®^; F. Hoffmann-La Roche, Basel, Switzerland; marketed in Japan by Chugai Pharmaceutical, Tokyo, Japan) at 6 mg/0.05 mL using a standard sterile technique. Treatment-naïve eyes received an initial loading phase consisting of three consecutive monthly injections, after which patients were transitioned to a modified treat-and-extend or pro re nata regimen guided by OCT findings and clinical response. Among the eyes managed under a treat-and-extend regimen, the maximum interval between consecutive faricimab injections reached the protocol-defined ceiling of 16 weeks. Eyes managed under a PRN regimen were re-treated as needed based on disease activity and had no predefined maximum injection interval. Re-injection was triggered by persistence or recurrence of intraretinal or subretinal fluid on SD-OCT. Eyes that had been switched from prior anti-VEGF therapy were treated according to individualized regimens at the discretion of the attending physician. Decisions to discontinue or switch therapy were based on the occurrence of adverse events or an inadequate response, defined as either an inadequate anatomical response (less than 10% reduction in central retinal thickness from baseline despite at least three consecutive monthly injections).

### 2.4. Outcome Measures

Demographic and systemic data—age, sex, glycated hemoglobin (HbA1c), serum creatinine, estimated glomerular filtration rate (eGFR), duration of diabetes, urinary albumin–creatinine ratio, lens status, interval from PPV to first IVF, and prior anti-VEGF exposure—were extracted from medical records. DME morphology was classified on SD-OCT as cystoid macular edema (CME), spongiform (diffuse retinal thickening), or serous retinal detachment. The primary outcome was the change in CRT from BL to M6 and M12. Secondary outcomes were the change in best-corrected visual acuity (BCVA, expressed as logMAR), the number of IVF injections during the 12-month period, and adverse events including intraocular inflammation. Baseline characteristics are reported for the full cohort of 12 eyes that received at least one IVF injection. Longitudinal anatomical and functional outcomes at M6 and M12 (CRT and BCVA) were analyzed in the 10 eyes that continued IVF through the entire 12-month follow-up period, whereas the 2 eyes that discontinued faricimab during the follow-up are described separately under treatment modifications.

### 2.5. Statistical Analysis

Continuous variables are expressed as mean ± standard deviation. Changes in CRT and BCVA across BL, M6, and M12 in eyes that continued IVF were analyzed using one-way repeated-measures analysis of variance (RM ANOVA) with Dunnett’s multiple comparisons test (using baseline as the control). A two-sided *p*-value < 0.05 was considered statistically significant. Statistical analyses and graphical presentations were performed using GraphPad Prism (version 10; GraphPad Software, Boston, MA, USA).

## 3. Results

### 3.1. Patient Characteristics

Twelve eyes of 11 patients (7 male, 4 female; mean age 59.4 ± 10.5 years) met the inclusion criteria. Mean HbA1c was 7.06 ± 0.63%, mean serum creatinine was 1.30 ± 0.84 mg/dL, mean eGFR was 62.3 ± 23.5 mL/min/1.73 m^2^, and the mean duration of diabetes was 14.9 ± 15.6 years. The mean interval from PPV to the first IVF was 25.6 ± 23.3 months. All eyes were pseudophakic. No eyes underwent Nd:YAG laser capsulotomy during the 12-month follow-up period. Eight eyes (66.7%) were treatment-naïve to anti-VEGF therapy, and 4 eyes (33.3%) had been switched to faricimab from prior anti-VEGF agents (aflibercept 2 mg in 3 eyes; brolucizumab in 1 eye) because of inadequate response. DME morphology at BL was CME in 7 eyes (58.3%) and spongiform in 5 eyes (41.7%). Detailed baseline characteristics are summarized in Table 1. Systemic management of diabetes mellitus and blood pressure was continued at the discretion of the patient’s primary care physician throughout the follow-up period; serial follow-up data on HbA1c and blood pressure were not centrally collected for the purposes of this retrospective analysis and are therefore not reported.

### 3.2. Anatomical Outcomes

**Figure 1:**
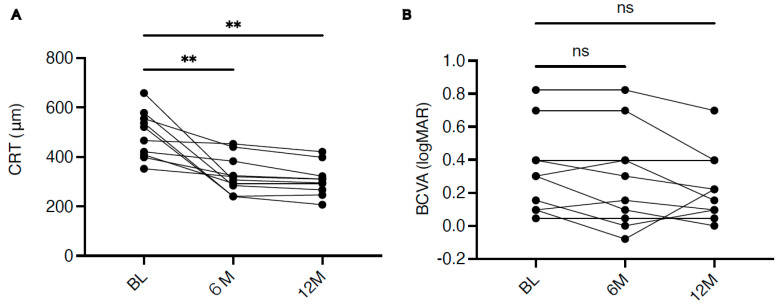
Changes in central retinal thickness (CRT) and best-corrected visual acuity (BCVA) over 12 months in 10 vitrectomized eyes that continued intravitreal faricimab. (**A**) CRT (µm) significantly decreased from baseline (BL) to 6 months (M6) and to 12 months (M12) (** *p* < 0.01, repeated-measures one-way ANOVA with Dunnett’s multiple comparisons test (using baseline as the control)). (**B**) BCVA (logMAR) showed a numerical improvement at M6 and M12, but the change did not reach statistical significance (ns, not significant). Each line represents an individual eye.

In the 10 eyes that continued IVF through 12 months, mean CRT decreased significantly from 489.5 ± 95.9 µm at BL to 328.5 ± 74.7 µm at M6 (mean reduction −161 µm; *p* = 0.0065) and to 307.0 ± 64.3 µm at M12 (mean reduction −182 µm; *p* = 0.0023) by repeated-measures ANOVA with Dunnett’s multiple comparisons test (Figure 1A). Reduction in CRT was observed in all 10 eyes at M12. The mean number of IVF injections during the 12-month period was 4.1 ± 1.9 (range 1–8), corresponding to an average of approximately 0.34 injections per month.

### 3.3. Functional Outcomes

Mean BCVA changed numerically from 0.33 ± 0.26 logMAR at BL to 0.28 ± 0.30 logMAR at M6 and 0.23 ± 0.21 logMAR at M12; however, the change did not reach statistical significance (*p* = 0.0894 for BL vs. M12). Visual acuity was maintained (within ±0.1 logMAR of baseline) or numerically better in 9 of 10 eyes (90%) at M12 (Figure 1B).

### 3.4. Treatment Modifications and Safety

Two eyes (16.7%) required a change in therapy. One eye, which initially showed a favorable anatomical response, developed intraocular inflammation, characterized by anterior chamber inflammation and vitritis without retinal vasculitis, after the third injection of faricimab; the inflammation resolved with topical corticosteroids and the patient was switched to aflibercept. The second eye showed an inadequate anatomical response after three IVF injections and was switched to brolucizumab combined with sub-Tenon triamcinolone, which resulted in adequate edema control.

### 3.5. Illustrative Cases

Case 1 (treatment-naïve, female, 57 years). A pseudophakic vitrectomized eye with cystoid DME (BL CRT 549 µm; BCVA 0.40 logMAR [Snellen 20/50]) showed marked anatomical improvement after the first IVF (CRT 234 µm at 1 month) that was sustained through 12 months (CRT 246 µm; BCVA 0.22 logMAR [Snellen 20/32]). The eye was treated with three IVF injections during the 12-month period.

Case 2 (treatment-naïve, male, 55 years). A vitrectomized eye with cystoid DME (BL CRT 578 µm; BCVA 0.30 logMAR [Snellen 20/40]) demonstrated rapid anatomical and functional improvement (CRT 346 µm at 1 month; CRT 267 µm and BCVA 0.00 logMAR [Snellen 20/20] at 12 months) after four IVF injections.

Case 3 (treatment-naïve, male, 61 years). A vitrectomized eye with spongiform DME (BL CRT 555 µm; BCVA 0.15 logMAR [Snellen 20/30]) required nine IVF injections within 12 months, reflecting a more refractory phenotype, but achieved CRT 406 µm and BCVA 0.10 logMAR [Snellen 20/25] at 12 months.

**Figure 2:**
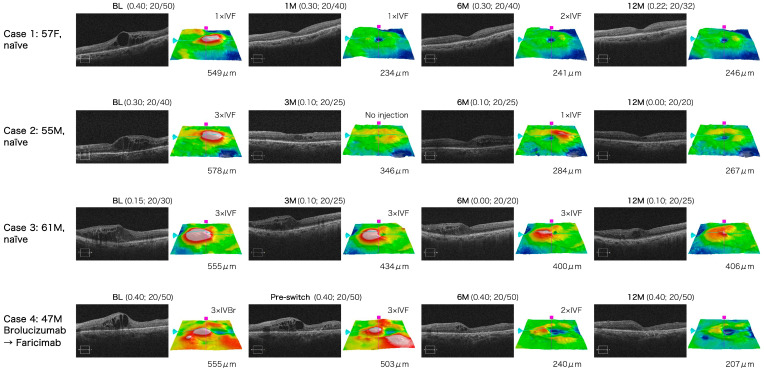
Representative ultra-widefield color fundus photographs and spectral-domain optical coherence tomography (SD-OCT) images of the four illustrative cases. The numbers above each OCT image (e.g., 1 × IVF, 3 × IVBr) indicate the cumulative number of injections administered between the preceding and the current time point. BCVA values shown in parentheses are expressed as logMAR with the Snellen equivalent (logMAR; Snellen). IVF, intravitreal faricimab; IVBr, intravitreal brolucizumab. Case 1 (57-year-old female, treatment-naïve): central retinal thickness (CRT) decreased from 549 µm at baseline (BL) to 246 µm at 12 months (M12). Case 2 (55-year-old male, treatment-naïve): CRT decreased from 578 µm to 267 µm with best-corrected visual acuity (BCVA) improving from 0.30 logMAR (Snellen 20/40) to 0.00 logMAR (Snellen 20/20). Case 3 (61-year-old male, treatment-naïve): CRT decreased from 555 µm to 406 µm. Case 4 (47-year-old male, switched from brolucizumab): CRT decreased from 555 µm at the time of switch (after three brolucizumab injections, BL of faricimab 503 µm) to 207 µm at M12 of faricimab therapy.

Case 4 (switched, male, 47 years). A vitrectomized eye with persistent cystoid DME despite three injections of brolucizumab was switched to faricimab. CRT was 555 µm immediately prior to the third (final) brolucizumab injection and 503 µm at the time of the first faricimab injection (i.e., the faricimab baseline; Day 0). CRT subsequently improved progressively to 240 µm at 6 months and 207 µm at 12 months while maintaining BCVA 0.40 logMAR (Snellen 20/50) (Figure 2).

Representative SD-OCT and fundus images for the four illustrative cases are provided in Figure 2.

## 4. Discussion

This study demonstrates that intravitreal faricimab achieves significant and durable reduction in macular thickness while maintaining stable visual acuity in vitrectomized eyes with DME after PPV for PDR over a 12-month period, and with a relatively low injection burden of approximately four injections per year. To our knowledge, this is the first dedicated study specifically examining the 12-month outcomes of intravitreal faricimab for DME in vitrectomized eyes following PPV for PDR. Although a small subgroup of vitrectomized eyes has been analyzed within one previous mixed cohort [8], the majority of large-cohort and long-term real-world studies of faricimab for DME have either excluded eyes with prior vitrectomy or have not separately analyzed this subgroup [9,10,11]. As a result, longer-term, dedicated outcome data in this clinically challenging population have been lacking. The only previous report focused specifically on faricimab in a vitrectomized eye is a single case report in neovascular age-related macular degeneration [13].

Vitrectomized eyes pose a recognized challenge for intravitreal pharmacotherapy. Removal of the vitreous gel reduces vitreous viscosity and increases drug clearance, shortening the intravitreal half-life of conventional anti-VEGF agents and potentially compromising drug durability [7]. In real-world practice, vitrectomized eyes with DME have been reported to require more frequent injections compared with non-vitrectomized eyes. Kusuhara et al. reported short-term outcomes of faricimab for DME in 21 eyes, in which 5 eyes (24%) had a history of prior vitrectomy; the mean injection frequency was 0.6/month in vitrectomized eyes versus 0.3/month in non-vitrectomized eyes during a mean follow-up of approximately 5.5 months [8]. The Japan Clinical REtina Study Group (J-CREST) recently reported real-world outcomes of faricimab for DME in 214 eyes from 13 Japanese centers, in which 18% of eyes had a history of prior vitrectomy, but did not analyze these vitrectomized eyes as a separate subgroup [9]. In contrast, in the present 12-month study, the mean injection frequency in vitrectomized eyes was approximately 0.34/month—comparable to that reported for non-vitrectomized eyes treated with faricimab. This finding suggests that simultaneous inhibition of VEGF-A and Ang-2 by faricimab may contribute to disease stabilization beyond what is achievable with VEGF-A blockade alone, potentially mitigating the durability disadvantage observed with conventional anti-VEGF agents in vitrectomized eyes. Our institution previously reported short-term (one-month) real-world outcomes of faricimab for DME in an unselected DME cohort at a separate Juntendo University hospital [12]; however, that earlier report did not analyze vitrectomized eyes separately and was limited to a one-month observation, with no patient overlap between the two cohorts. By specifically focusing on a dedicated cohort of vitrectomized eyes followed for 12 months, the present study extends and complements this earlier work and addresses a distinct, clinically relevant gap in the existing real-world evidence.

Faricimab also has physical properties that may be particularly favorable in vitrectomized eyes. Its molecular weight (~150 kDa) is greater than that of aflibercept (~115 kDa), which may translate into slower intravitreal clearance in the absence of the vitreous barrier. Chujo et al. recently reported a vitrectomized eye with neovascular age-related macular degeneration that was refractory to multiple anti-VEGF agents but responded uniquely to faricimab, attributing the response in part to altered intravitreal drug clearance in vitrectomized eyes and faricimab’s prolonged activity [13]. In our series, all four switched eyes demonstrated anatomical improvement after switching to faricimab, consistent with recent meta-analytic and review evidence supporting switching to faricimab in eyes with DME refractory to prior anti-VEGF therapy [15,16]. These findings support the hypothesis that the pharmacokinetic and pharmacodynamic properties of faricimab may translate into clinical benefit in vitrectomized eyes that have responded suboptimally to other anti-VEGF agents.

In contrast to the significant anatomical improvement, mean BCVA remained essentially stable over 12 months (*p* = 0.0894 at M12). The lack of measurable visual gain is not unexpected in this cohort: all eyes had previously undergone PPV for PDR, an advanced stage of diabetic retinal disease in which diabetic macular ischemia [17] and diabetic retinal neurodegeneration [18] have likely already progressed, both of which are well-recognized factors that limit visual recovery despite successful resolution of macular edema. Stable BCVA together with substantial and durable CRT reduction is therefore a clinically meaningful outcome in this advanced disease setting.

Notably, despite the relatively long mean diabetes duration (14.9 years), advanced disease background (all eyes had previously required PPV for PDR), and chronic kidney disease in some patients, only one eye (8.3%) showed inadequate anatomical response requiring a switch in therapy. The other discontinuation was related to intraocular inflammation manifesting as anterior chamber inflammation and vitritis without retinal vasculitis, a known but rare adverse event of faricimab [2,3], which resolved without sequelae. Our cohort showed substantial heterogeneity in diabetes duration (range, approximately 1–55 years) and in the interval between the index PPV and the first faricimab injection (mean 25.6 ± 23.3 months). This heterogeneity likely reflects the real-world nature of clinical practice and may have contributed to the variability in functional response observed across individual eyes; nonetheless, the consistent anatomical improvement across this heterogeneous group suggests a robust treatment effect of faricimab on macular edema, even in eyes with widely varying disease chronicity.

Several limitations should be acknowledged. This was a single-center, retrospective study with a small sample size (12 eyes of 11 patients), which substantially limits statistical power; in particular, the numerical improvement in BCVA did not reach statistical significance (*p* = 0.0894). The absence of a concurrent control group (e.g., vitrectomized eyes treated with aflibercept, ranibizumab, or brolucizumab) prevents any direct conclusion regarding the superiority of faricimab over alternative anti-VEGF agents in this setting. Treatment regimens were not standardized, reflecting real-world practice. Quantitative measures of retinal and macular ischemia (e.g., wide-field fluorescein angiography or OCT angiography metrics, including foveal avascular zone area and parafoveal vessel density) were not systematically analyzed; given that diabetic macular ischemia is an established determinant of visual outcomes in DME [17], the absence of these data is a particularly important limitation when interpreting the lack of significant BCVA improvement and may, at least in part, account for the heterogeneous functional responses observed despite consistent anatomical improvement. Larger, ideally prospective and comparative trials—including head-to-head comparisons with other anti-VEGF agents in vitrectomized eyes—are warranted to confirm the long-term efficacy and safety of faricimab and to clarify whether dual VEGF-A/Ang-2 blockade offers a pharmacologic advantage over VEGF-A monotherapy in this population.

## 5. Conclusions

In this first dedicated 12-month study of intravitreal faricimab for DME in eyes vitrectomized for PDR, faricimab significantly improved central retinal thickness while maintaining stable visual acuity, with a low injection burden comparable to that reported in non-vitrectomized eyes. These findings suggest that faricimab represents a valuable therapeutic option for DME in vitrectomized eyes—a population that has been largely excluded from large-cohort real-world studies and in which the pharmacokinetic limitations of intravitreal anti-VEGF therapy have historically been a clinical challenge.

## Figures and Tables

**Table 1:** Baseline characteristics of 12 vitrectomized eyes with DME treated with intravitreal faricimab.

Variable	Value (n = 12 Eyes/11 Patients)
Age (years)	59.4 ± 10.5
Sex (male/female)	7/4
HbA1c (%)	7.06 ± 0.63
Serum creatinine (mg/dL)	1.30 ± 0.84
eGFR (mL/min/1.73 m^2^)	62.3 ± 23.5
Duration of diabetes (years)	14.9 ± 15.6
Interval from PPV to first IVF (months)	25.6 ± 23.3
Lens status (pseudophakic)	12 (100%)
Treatment status (naïve/switched)	8/4
DME morphology (CME/spongiform)	7/5
Baseline CRT (µm)	486.1 ± 87.3
Baseline BCVA (logMAR)	0.32 ± 0.24

Values are expressed as mean ± standard deviation or n (%). BCVA, best-corrected visual acuity; CME, cystoid macular edema; CRT, central retinal thickness; DME, diabetic macular edema; eGFR, estimated glomerular filtration rate; IVF, intravitreal faricimab; PPV, pars plana vitrectomy.

## Data Availability

The data presented in this study are available on reasonable request from the corresponding author. The data are not publicly available due to privacy and ethical restrictions.
